# Conflicts of Interest: Gulson’s Response

**Published:** 2004-10

**Authors:** Brian Gulson

**Affiliations:** Graduate School of the Environment Macquarie University Sydney, New South Wales, Australia E-mail: bgulson@gse.mq.edu.au

We commend the Center for Science in the Public Interest (CSPI) for their investigation into conflicts of interest statements in leading scientific and medical journals, but we take exception to our inclusion (Gulson et al. 2004) as an example of providing misinformation to *EHP*.

Goozner’s sweeping statement that my colleague had previously received research funding, compensation, or stood to gain financially from Pasminco Ltd. is highly inaccurate. We received no research funding, compensation, or financial gain from the company to undertake this study. In fact, if Goozner had read even the abstract of our article, he would have noted that the findings were detrimental to the company, as the dominant source of lead in the environment and children probably derived from smelter emissions. Furthermore, the smelter closed in September 2003 and the company no longer exists. With respect to the association of my colleague, Karen Mizon, to her husband’s company and the (consulting) work undertaken for Pasminco Ltd., the work [an International Organization for Standardization (ISO) Guide 25 accreditation assessment of the company’s on-site laboratory (ISO/International Electrotechnical Commission 1990)] was undertaken by the owner for the accreditation body while he was employed by a federal government research organization, and he was not paid for this audit.
